# *Candida albicans* Induces Metabolic Reprogramming in Human NK Cells and Responds to Perforin with a Zinc Depletion Response

**DOI:** 10.3389/fmicb.2016.00750

**Published:** 2016-05-19

**Authors:** Daniela Hellwig, Jessica Voigt, Maria Bouzani, Jürgen Löffler, Daniela Albrecht-Eckardt, Michael Weber, Sascha Brunke, Ronny Martin, Oliver Kurzai, Kerstin Hünniger

**Affiliations:** ^1^Septomics Research Center, Leibniz Institute for Natural Product Research and Infection Biology – Hans Knoell Institute and Friedrich Schiller UniversityJena, Germany; ^2^Medizinische Klinik und Poliklinik II, Universitätsklinikum WürzburgWürzburg, Germany; ^3^BioControl Jena GmbHJena, Germany; ^4^Department Microbial Pathogenicity Mechanisms, Leibniz Institute for Natural Product Research and Infection Biology – Hans Knoell InstituteJena, Germany

**Keywords:** *Candida albicans*, NK cells, perforin, glycolysis, zinc

## Abstract

As part of the innate immune system, natural killer (NK) cells are directly involved in the response to fungal infections. Perforin has been identified as the major effector molecule acting against many fungal pathogens. While several studies have shown that perforin mediated fungicidal effects can contribute to fungal clearance, neither the activation of NK cells by fungal pathogens nor the effects of perforin on fungal cells are well-understood. In a dual approach, we have studied the global gene expression pattern of primary and cytokine activated NK cells after co-incubation with *Candida albicans* and the transcriptomic adaptation of *C. albicans* to perforin exposure. NK cells responded to the fungal pathogen with an up-regulation of genes involved in immune signaling and release of cytokines. Furthermore, we observed a pronounced increase of genes involved in glycolysis and glycolysis inhibitor 2-deoxy-D-glucose impaired *C. albicans* induced NK cell activation. This strongly indicates that metabolic adaptation is a major part of the NK cell response to *C. albicans* infections. In the fungal pathogen, perforin induced a strong up-regulation of several fungal genes involved in the zinc depletion response, such as *PRA1* and *ZRT1*. These data suggest that fungal zinc homeostasis is linked to the reaction to perforin secreted by NK cells. However, deletion mutants in *PRA1* and *ZRT1* did not show altered susceptibility to perforin.

## Introduction

Natural killer (NK) cells are CD56^+^ CD3^-^ cytotoxic lymphocytes of the innate immune system. These cells exert important antiviral and antitumoral activity by killing virus-infected or cancerous cells. Major effector mechanisms are the release of transmembrane pore forming perforin and granzyme B or induction of death receptor-mediated apoptosis (Lanier, 2008). Activation of NK cells is controlled by activating and inhibiting germline-encoded receptors. Classically, activation by virus-infected or cancerous cells occurs via *missing self*-signaling, i.e., the absence of constitutively expressed markers like MHC class I on the surface of target cells that results in abrogation of signaling by inhibiting receptors and thus a dominance of activating signals (Lanier, 2008). In addition, activating receptors may recognize pathogen or cell stress-induced ligands (Martinet and Smyth, 2015). Accumulating evidence suggests that NK cells are directly involved in the response to fungal infections (Schmidt et al., 2013). Beside data from murine models, indicating that NK cells are required for full protection against *Aspergillus fumigatus*, *Cryptococcus neoformans* and – depending on the immunological status – *Candida albicans*, patients with a NK cell defect due to primary hemophagocytic lymphohistiocytosis are predisposed to fungal infection (Lipscomb et al., 1987; Sung et al., 2001; Morrison et al., 2003; Albisetti et al., 2004; Li et al., 2013; Quintin et al., 2014). NK cells exhibit direct antifungal activity against *C. neoformans* and enhance clearance of this pathogen *in vivo* (Lipscomb et al., 1987; Levitz et al., 1994; Ma et al., 2004; Schmidt et al., 2013). The antifungal activity against *C. neoformans* is mainly mediated via perforin (Ma et al., 2004). In addition to *C. neoformans*, antifungal activity of NK cells has also been demonstrated for *Paracoccidioides brasiliensis*, *Coccidioides immitis*, *Rhizopus arrhizus (Rhizopus oryzae)*, *A. fumigatus* and *C. albicans* (Jimenez and Murphy, 1984; Petkus and Baum, 1987; Bouzani et al., 2011; Schmidt et al., 2011, 2015; Voigt et al., 2014). We and others have shown that human NK cells are activated by *A. fumigatus* and show fungicidal activity against this mold pathogen. The molecular mechanisms are not yet fully understood but evidence suggests that beside perforin, also IFN-γ may contribute to fungal damage (Bouzani et al., 2011; Schmidt et al., 2011). *C. albicans* has been shown to activate NK cells in a contact dependent manner, which may even result in phagocytic uptake of the fungal pathogen (Voigt et al., 2014). *C. albicans* mediated activation of NK cells triggers degranulation of secretory granules and the release of several cytokines including IFN-γ and TNF-α. Furthermore, NK cells show immunomodulatory potential in alliance with neutrophilic granulocytes (Voigt et al., 2014). Recently, NKp30 has been identified as a receptor for fungal pathogens on human NK cells (Li et al., 2013). Perforin also appears to be the major mediator of anti-*Candida* activity in human NK cells (Voigt et al., 2014). Taken together, studies performed with different fungal pathogens and both human and murine NK cells indicate that perforin is the major antifungal effector of NK cells. Perforin is stored in secretory granules of cytotoxic lymphocytes and can be released into an immunological synapse. In the target cell membrane, perforin oligomerizes and forms large transmembrane pores, which penetrate and damage the target cell membrane. In perforin-granzyme mediated cell death, granzyme enters the target cell via these pores and initiates apoptosis in the target cell (Voskoboinik et al., 2015).

To further elucidate the interaction of human NK cells and *C. albicans*, we performed an array-based analysis of the transcriptional response of NK cells after confrontation with *C. albicans* as well as of *C. albicans* after exposure to perforin. Transcriptome analyses have provided fundamental insights into host pathogen interaction: the response of pathogens to host cells and tissue offers insight into the mechanisms required to invade the host and successfully evade host immunity. On the other hand the transcriptional response of host cells to infection enables the identification of cellular defense mechanisms and patterns of activation (Waddell et al., 2007). Our results show that beside functional activation of human NK cells, *C. albicans* also induces an activation-specific metabolic shift, namely enhanced glycolytic activity. The exposure of *C. albicans* to perforin triggered a narrow set of regulated genes. Most importantly, perforin induced a prototypic zinc depletion response. Despite this, deletion of genes essential for zinc acquisition did not alter effects of perforin on *C. albicans*.

## Materials and Methods

### Fungal Strains and Culture Conditions

The *C. albicans* wildtype strain SC5314 (Gillum et al., 1984) and the mutants *pra1*Δ (ura3:: imm434/ura3:: imm43, his1::hisG/his1::hisG, *pra1*::*HIS1*/*pra1*::*ARG4*+CIp10, Citiulo et al., 2012) and *zrt1*Δ (*ura3:: imm434/ura3:: imm434, his1::hisG/his1::hisG, zrt1::HIS1/zrt1::ARG4*+CIp10, Citiulo et al., 2012) were used in this study. For co-incubation with NK cells, strains were cultured overnight in standard YPD medium (1% yeast extract, 2% bacto-peptone, 2% D-glucose) at 30°C and diluted 1:10 in fresh medium for a 1.5 h induction culture at 30°C on a shaker prior to the experiments. Harvested cells were washed three times in PBS and resuspended in Stem Cell Growth Medium (SCGM, Cell Genix) + 10% human serum (PAA). To obtain *C. albicans* filaments, cells were grown overnight in M199 medium (9.8 g/l M199, 35.7 g/l HEPES, 2.2 g/l NaHCO_3_), pH 4 at 37°C to stationary phase in a shaking incubator. Fungal cells were then reseeded in M199 medium, pH 8 and cultured for approximately 1 h at 37°C, which induced filamentous growth in *C. albicans*. Filaments were killed by incubation in 0.1% thimerosal (Sigma-Aldrich) in HBSS at 37°C for 1 h and then rinsed extensively. For co-incubation of *C. albicans* with perforin, fungi were suspended in RPMI1640 (Biochrom) containing 5% heat-inactivated FBS (Sigma-Aldrich) or SD-*N*-acetyl-D-glucosamine media [0.67% YNB w/o amino acids (Sigma-Aldrich), 2% *N*-acetyl-D-glucosamine] to a final concentration of 4 × 10^4^ cells/ml. 100 μl per well were seeded into lumitrac 96-well plates (Greiner). Hyphal induction occurred in the absence (control) or presence of different perforin concentrations (Enzo, isolated from YT cell line) for 1–8 h at 37°C and 5% CO_2_. Concentrations of perforin ranged from 100 to 500 ng/ml.

### Human Natural Killer Cells

Primary human PBMCs were isolated from the buffy coat of healthy donors by standard Ficoll gradient centrifugation (Biochrom AG). Untouched NK cells were separated by MACS technology using the NK cell isolation kit (Miltenyi Biotec) according to manufacturer’s instructions. Purity of the cells was checked by FACS analysis with results of >95% CD56^+^, CD16^+^, CD3^-^, CD14^-^ NK cells. Freshly isolated human NK cells were cultivated at a concentration of 2 × 10^6^/ml in 1 ml Stem Cell Growth Medium (SCGM; Cell Genix) containing 10% human serum (PAA) and 100 U/mL interleukin-2 (IL-2, Immunotools) in 24 well plates. Primary NK cells were used at day 1 after isolation. To generate cytokine activated NK cells, half of the medium was replaced after 3 days of cultivation by fresh SCGM containing 10% human serum supplemented with 100 U/ml IL-2, 50 ng/ml IL-15, 1000 U/ml IFN-α, and 2000 U/ml IFN-β (Immunotools) and cultivated for another 3 days. This study was carried out in accordance with the Declaration of Helsinki. All protocols involving human blood donors were approved by the local Ethics Committee (permit number: 3639-12/12).

### Confrontation Assay

Natural killer cells were either co-cultured with *C. albicans* yeasts at a MOI of 0.5 for 4 h (fungal viability assay, XTT assay) or at a MOI of 1 for 3 and 6 h (transcriptome analysis) at 37°C and 5% CO_2_ in SCGM medium containing 10% human serum in reaction tubes. For glycolysis inhibitor treatment, NK cells were pre-incubated with 100 mM 2-deoxy-D-glucose (2-DG, Sigma-Aldrich) for 30 min at 37°C and 5% CO_2_ and further treated with 100 mM 2-DG during confrontation with thimerosal-killed *C. albicans* filaments (MOI of 0.5) for 4 h.

### Flow Cytometry

Expression of NK cell surface markers was analyzed via differential FACS staining and subsequent measurement with the FACSCanto II (BD). Changes in surface expression were investigated for Fcγ receptor III (mouse anti-human CD16-APC, clone 3G8) and degranulation marker CD107a (mouse anti-human CD107a-PE, clone H4A3) as described previously (Voigt et al., 2014).

FlowJo 7.6.4 software was used for analysis.

### Quantification of Secreted Proteins

The concentrations of secreted proteins within the supernatant of confrontation samples were determined using Luminex technology [MILLIPLEX MAP Human CD8+ T Cell Magnetic Bead Panel (Perforin, GM-CSF, IFN-γ, TNF-α); Millipore]. The analysis was performed according to the instructions from the manufacturer.

### Fungal Viability Assay

Killing of *C. albicans* was determined by XTT assay. *C. albicans* in medium alone (control) and NK cell – *Candida* co-cultures were washed with cold ddH_2_O and 0.2% Triton X to disrupt the immune cells and *Candida* cells were pelleted by centrifugation at 14.000 × *g* for 5 min at 4°C. Afterwards, the fungal cell pellet was resuspended in a solution of 0.5 mg/ml XTT (Sigma-Aldrich) heated at 55°C in PBS for 30 min and supplemented with 50 μg/ml Coenzyme Q (Sigma-Aldrich). Following an incubation step of 1 h at 37°C, the absorption of the cell-free supernatant was measured at 450 nm wavelength (reference at 650 nm). The fungal killing was calculated according to the formula: percentage of killing = (1-X/Y) x 100. X stands for the absorption of the different culture conditions and Y for the absorption of *C. albicans* in medium alone (control, set to 100%).

### Microscopy and Image Analysis

After fixation [Histofix (Roth), 4%] and washing with PBS (Biochrom) fungal cells were stained with Calcofluor white (Sigma) for 10 min and washed three times with PBS. Images were taken with a Zeiss LSM780 confocal microscope and hyphal length and averaged relative fluorescence intensities were analyzed with ZEN 2012 software.

### Transcriptome Analysis of NK Cells

RNAprotect cell reagent (Qiagen) was added 1:5 to the experiments and samples were stored at -80°C for further treatment. RNA isolation was performed with RNAeasy Mini Kit (Quiagen) following the manufacturer’s instructions. RNA amplification and cRNA transcription was done with Illumina TotalPrep RNA Amplification Kit (Illumina) following the manufacturer’s instructions. HumanHT-12 v4 Expression BeadChip Kits (Illumina) were used for transcriptome analyses. After quality control, arrays were preprocessed using R software version 2.14.1^1^. Data were normalized using quantile normalization and logarithm. Probes were used only if they were present on at least three chips. A linear model was fit to the normalized data resulting in one normalized intensity value per transcript and chip. Transcripts were regarded as being significantly differentially expressed when they showed an absolute fold change of larger than 2 between at least two different conditions and an FDR adjusted *t*-test *p*-value of less than 0.05. Differentially regulated transcripts were categorized according to KEGG using DAVID^2^. Microarray raw data are available at Array Express (http://www.ebi.ac.uk/arrayexpress/ and accession number E-MTAB-4105).

### Transcriptome Analysis of *C. albicans*

The isolation of fungal RNA was done as described previously (Martin et al., 2011). Sample RNAs were labeled with Cy5-CTP and hybridized with a Cy3-labeled common reference RNA on *C. albicans* DNA microarrays (ClinEuroDiag, Brussels, Belgium). The slides were hybridized, washed and scanned with a Genepix 4000B (Molecular Devices) as described in a previous study (Martin et al., 2011). Arrays were preprocessed using R software version 2.14.1^1^ after a quality control. Normalization of the data was performed with Print-tip loess and Gquantile methods. A linear model was fit to the normalized data. Transcripts were only regarded as being significantly differentially expressed when they showed an absolute fold change of larger than 2 and an FDR-adjusted *t*-test *p*-value ≤ 0.05. For qRT-PCRs, 100 ng of total RNA were utilized with Brilliant III Ultra Fast SYBR Green qRT PCR Kit (Agilent Technologies). Expression analysis was performed on an Applied Stratagene Mx3005P (Agilent Technologies) and calculated by the ΔΔCt method (Pfaffl, 2001). Primers used in this study are shown in Supplementary Table S1. Microarray raw data are available at Array Express (http://www.ebi.ac.uk/arrayexpress/ and accession number E-MTAB-4109).

### Statistical Analyses

For all experiments, at least three independent replicates using NK cells from non-identical donors or *C. albicans* cells from non-identical cultures, respectively were used. Data are presented as arithmetic mean ± standard deviation (SD) and statistical significance was calculated using a two-sided Student’s *t*-test for unpaired data, shown as ^∗^*p* < 0.05, ^∗∗^*p* < 0.01.

## Results

### Transcriptional Dynamics of Primary and Cytokine Activated NK Cells during Interaction with *C. albicans* Reveal Functional and Metabolic Activation

**FIGURE 1:**
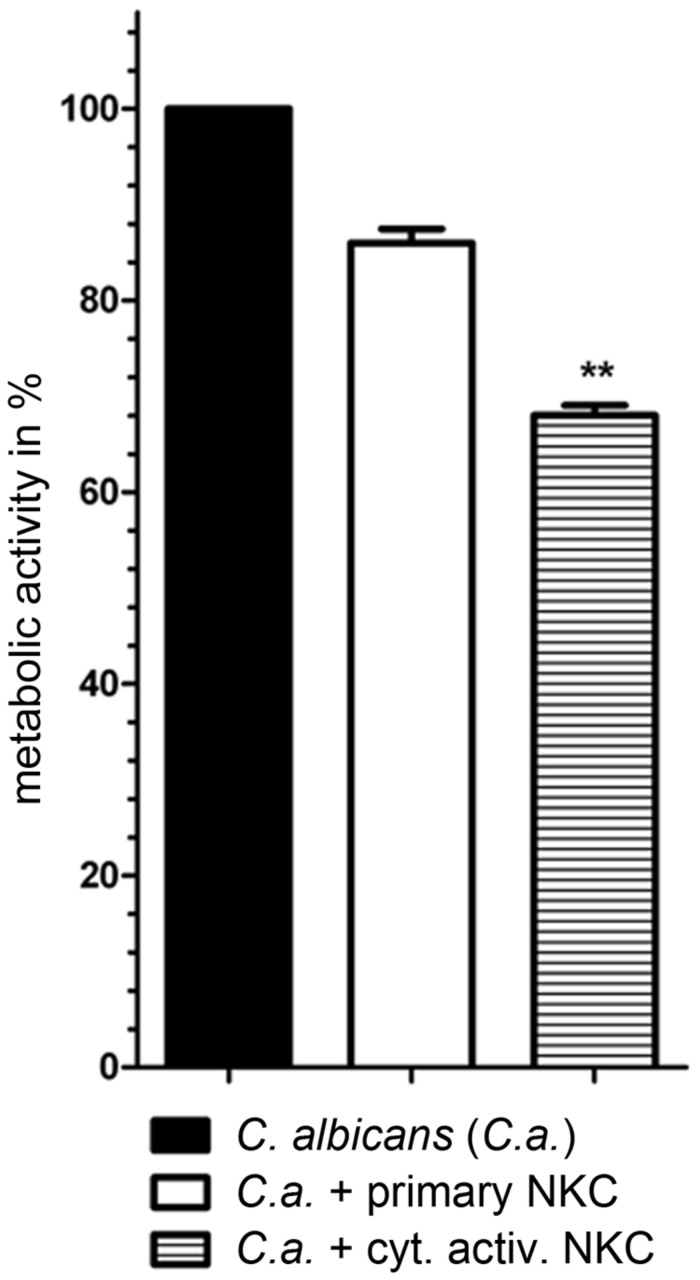
**Reduced metabolic activity of *Candida albicans* during confrontation with cytokine activated NK cells.** Fungal metabolic activity was determined by XTT assay. *C. albicans* wild type SC5314 was co-incubated with either primary or cytokine activated NK cells. Metabolic rates are given in percentage of *C. albicans* survival without presence of NK cells (set to 100%). The bars show mean ± standard deviation of at least three independent experiments with NK cells from different donors. Estimation of *p*-values was performed with unpaired, two-sided Student’s *t*-test and asterisks indicate significance, ^∗∗^*p* ≤ 0.01.

**FIGURE 2:**
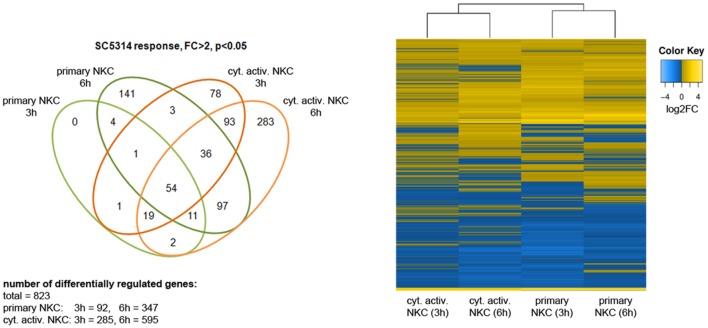
**Transcriptomic response of NK cells to *C. albicans*.** Prior to RNA isolation, primary and cytokine treated NK cells were incubated with *C. albicans* wild type for 3 and 6 h. We performed three independent experiments with NK cells from different donors. Venn diagram and heat map show the differentially expressed genes with a minimum absolute fold change —FC— of 2 in two different conditions (*p* < 0.05).

In previous analyses, cytokine activated NK cells had been used for confrontation with *C. albicans* due to their potential use in immunotherapy (Voigt et al., 2014). To quantify the effect of cytokine induced activation, we measured the inhibitory potential of both primary and cytokine activated NK cells on *C. albicans* metabolic activity. Only slight changes in metabolic activity of *C. albicans* could be observed in the presence of primary NK cells, while cytokine primed NK cells reduced fungal metabolic activity by 32% ± 10% (**Figure 1**, Voigt et al., 2014). To get a more detailed view on the differences between primary and cytokine activated NK cells during *C. albicans* confrontation, we investigated transcriptomic changes induced by *C. albicans* infection in both cell types. For this, primary and cytokine activated NK cells from three independent donors (three biological replicates) were co-incubated with *C. albicans*, harvested after 3 and 6 h of infection and analyzed on an Illumina HumanHT-12 v4 Expression BeadChip. Upon comparison of gene expression of NK cells confronted with *C. albicans* to that of mock-treated cells, a significantly higher number of genes was differentially regulated in cytokine primed NK cells, indicating a faster and more intense response compared to the primary cells (**Figure 2**; **Supplementary Table S2**). After 3 h of co-incubation, 92 genes were differentially regulated in primary NK cells, whereas 285 genes were differentially regulated in cytokine activated NK cells. At 6 h of infection these numbers increased to 347 genes (primary NK cells) and 595 genes (cytokine activated NK cells), respectively (**Figure 2**; **Supplementary Table S2**). A comparison of gene expression of both NK cell types revealed a core transcriptional response of 204 genes toward *C. albicans* that were differentially regulated in cytokine activated and primary NK cells (**Supplementary Table S2**). Pathway analysis revealed differential regulation of genes involved in processes like immune cell signaling pathways, cytokine–cytokine receptor interaction and glycolysis (**Table 1**). Genes involved in cellular signaling pathways were among the most up-regulated genes especially in cytokine activated NK cells. A prominent example is the MAPK signaling pathway, known to be a major cascade in innate immune activation. A closer look at the genes involved in the MAPK signaling pathway highlights a more rapid and more intense response of cytokine activated NK cells as the transcriptional regulation of the central regulator MYC is higher in comparison to primary NK cells (**Supplementary Table S2**). Strong up-regulation of the phosphatases DUSP2, 4, 5, and 6 indicates the necessity of down-regulation of MAPK signaling cascade due to strong activation.

**Table 1:** KEGG categories significantly overrepresented in differently expressed genes of primary and cytokine activated NK cells (NKC) challenged with *Candida albicans*.

KEGG	Term	# of genes	*p*-value	Genes
**(A) Primary and cytokine activated NKC (core response).**
Hsa00010	Glycolysis/gluconeogenesis	7	0.00014	LDHA, PGAM4, ALDOC, PGAM1, HK2, ENO2, PFKP
Hsa04060	Cytokine–cytokine receptor interaction	13	0.00017	CSF2, IL3, IL8, IL21R, CCL4L1, TNFSF9, CCL4, CCL14, CCL3L1, CX3CR1, IFNG, VEGFA, XCL1
**(B) Primary NKC specific response.**
Hsa04062	Chemokine signaling pathway	5	0.020	CXCL1, CCL3, IL8, CCR1, PRKACB
**(C) Cytokine activated NKC specific response.**
Hsa04110	Cell cycle	10	0.0015	E2F2, CDKN1A, GADD45G, TTK, CDK6, CDC20, CDC25C, GADD45B, MYC, CDC25B
Hsa04010	MAPK signaling pathway	15	0.0018	PRKCA, TNF, MAP2K3, FASLG, NR4A1, CDC25B, DUSP5, DUSP4, DUSP2, NTRK1, MAP3K8, GADD45G, GADD45B, MYC, DUSP6
Hsa00010	Glycolysis/gluconeogenesis	6	0.0095	GPI, PKM2, HK1, PGK1, GAPDHL6, ALDH3A2
Hsa04650	Natural killer cell mediated cytotoxicity	8	0.0255	PRKCA, ICAM1, CD244, TNF, PIK3CD, NFAT5, FASLG, NFATC1
Hsa04060	Cytokine–cytokine receptor interaction	12	0.02686	CCL1, LIF, TNFRSF9, TNF, TNFRSF12A, CLCF1, CXCL16, TNFSF14, FASLG, CCL4L2, KIT, LTA


#### Cytokine/Chemokine Signaling

**FIGURE 3:**
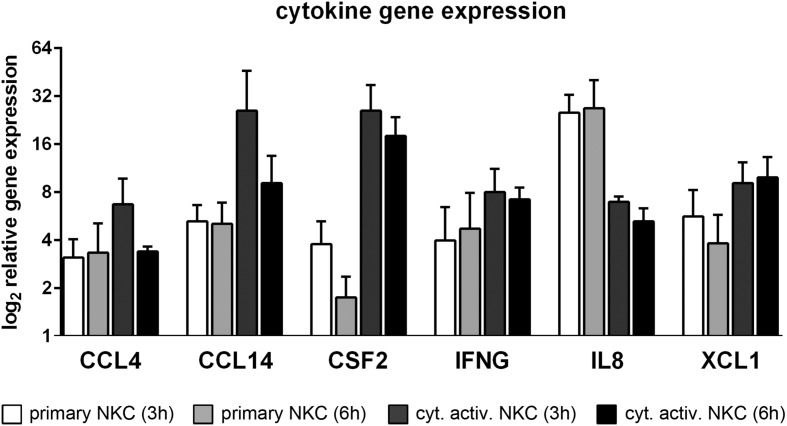
**Enhanced expression of cytokine genes in *C. albicans* infected NK cells.** Changes in transcript levels of cytokine genes are detected during co-incubation of primary and cytokine activated NK cells with *C. albicans* for 3 and 6 h. Data are displayed as fold change of *C. albicans*-treated cells relative to mock-treated cells (FC > 2, *p* < 0.05).

Important pro-inflammatory cytokines and chemokines have been quantified in the supernatant of cytokine activated NK cells after confrontation with *C. albicans* wild type in our previous study (Voigt et al., 2014). Here, the corresponding genes could be analyzed for their transcriptional regulation. Indeed, significant up-regulation of genes encoding for GM-CSF (CSF2), IFN-γ, MIP-1β (CCL4) in primary and cytokine activated NK cells could be detected on the transcriptomic level (**Figure 3**). In addition to this and among several other chemokine and cytokine encoding genes (**Figure 3**; **Table 1**), the genes for IL8, involved in recruitment and activation of neutrophils (Duggan et al., 2015), CCL14, a homolog of CCL4 known to activate monocytes (Schulz-Knappe et al., 1996) and XCL1, an activator of antigen-presenting dendritic cells (XCR1^+^ subpopulation) and promoter of a Th1 response (Lei and Takahama, 2012), were strongly up-regulated, further extending the important role of NK cells as modulators of other immune cells. This modulatory function is also demonstrated by the induction of the pre-prohormone of adrenomedullin (ADM; see **Supplementary Table S2**), which after proteolytic cleavage exerts several functions, including vasodilation and antimicrobial activity against several bacterial pathogens (Elsasser and Kahl, 2002).

#### Glycolysis

**FIGURE 4:**
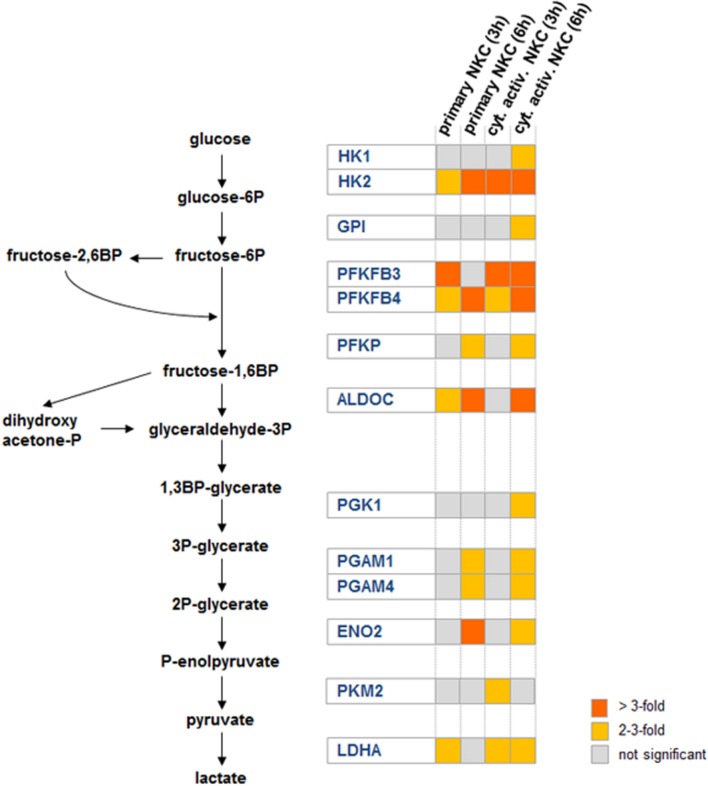
**Glycolytic reprogramming is a key feature of the NK cell response to *C. albicans.*** Intermediate metabolites of the glycolysis pathway are colored in black, while significantly up-regulated genes encoding for glycolytic enzymes are highlighted in blue. The heat map shows gene expression levels of the highlighted genes in the glycolysis pathway for both conditions (primary and cytokine activated NKC) for both time points (3 and 6 h).

**FIGURE 5:**
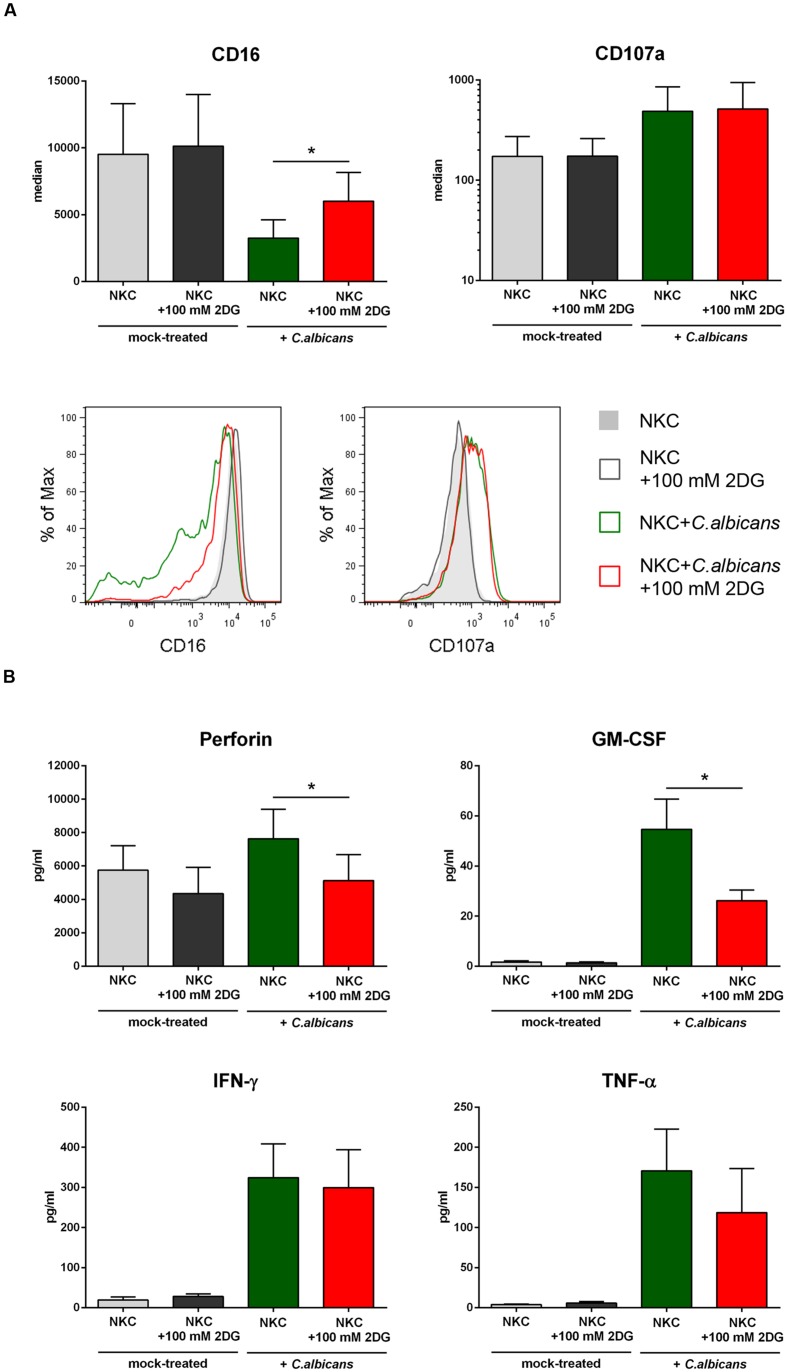
**Blockage of glycolysis has selectively effects on NK cell activation induced by *C. albicans.*** Human NK cells were either pre-incubated in media alone or treated with the glycolysis inhibitor 2-DG (100 mM) for 30 min prior to inoculation of *C. albicans* for 4 h to investigate the impact of glycolysis on NK cell activation. Samples were compared to mock-infected NK cells in the absence or presence of 100 mM 2-DG. **(A)** NK cell activation is shown 4 h after inoculation of *C. albicans* by changes in surface expression levels of the activation markers CD16 and CD107a. Light gray filled (non-treated) and black open histograms (2-DG-treated) indicate basal expression on NK cells from mock-infected samples. Green open histograms indicate surface levels on *C. albicans*-infected NK cells in the absence of 2-DG, red open histograms show 2-DG-treated NK cells following *C. albicans* infection. Data from one of seven independent experiments with virtually identical results are shown. Quantitative analysis was performed for both surface markers. Data shown are mean ± standard deviation. CD16 revealed significantly different surface levels on NK cells after contact with *C. albicans* when glycolysis was blocked (red bar). **(B)** Supernatants of NK cell infection experiments were analyzed for secretion of perforin, GM-CSF, IFN-γ and TNF-α. Mock-infected NK cells in the absence (light gray bars) and presence of 2-DG (dark gray bars) were compared to *C. albicans*-infected NK cells without (green bars) and with glycolysis blockage (red bars). Bars show mean ± standard deviation of at least three independent experiments with NK cells from different donors. Estimation of *p-*values was performed with unpaired, two-sided Student’s *t-*test and asterisks indicate significance, ^∗^*p* ≤ 0.05.

A shift to aerobic glycolysis has been described as a metabolic hallmark of NK cell activation (Donnelly et al., 2014). In our data, we observed an up-regulation of several genes involved in glycolysis (**Figure 4**). These genes were either up-regulated in both types of NK cells or in cytokine activated NK cells only (**Supplementary Table S2**). Within the category glycolysis, the hexokinase 2 encoding gene HK2, one of the key rate limiting enzymes in glycolysis, was the most up-regulated gene (**Supplementary Table S2**). In addition, other important checkpoints in glycolysis, namely phosphofructokinase (gene PFKP up-regulated in primary and cytokine activated NK cells at 6 h *post-infection*) and lactatedehydrogenase (gene LDHA, up-regulated in primary and primed NKC at 3 h and 6 h) were up-regulated. Furthermore two bifunctional kinases/phosphatases that regulate the concentration of the glycolytic byproduct and activator of phosphofructokinase fructose-2,6-bisphosphate (PFKFB3, PFKFB4) were strongly up-regulated in primary and cytokine activated NK cells. Taken together, these data suggest a strong metabolic reprogramming of NK cells toward glycolysis after contact with *C. albicans*. To determine the contribution of glycolysis to NK cell antifungal activity, NK cells were treated with the glycolysis inhibitor 2-deoxy-D-glucose (2-DG) throughout infection with *C. albicans*. Since 2-DG was found to significantly diminish *C. albicans* filamentation in media alone (data not shown) and filamentation is a prerequisite for NK cell activation, glycolysis inhibitor assays were performed using thimerosal-killed *C. albicans* filaments. Incubation of NK cells with 2-DG clearly prevented CD16 down-regulation and significantly reduced the secretion of perforin and GM-CSF by NK cells after contact with *C. albicans* (**Figure 5**). In addition, TNF-α level in response to *C. albicans* was reduced after 2-DG treatment, although this effect was not statistically significant. The effects of 2-DG treatment were specific, as increased surface exposure of degranulation marker CD107a and release of IFN-γ induced by *C. albicans* showed the same patterns in the absence or presence of 2-DG and were therefore not affected by the glycolysis blockage.

### Perforin Inhibits Filamentation of *C. albicans* in a Time and Dose Dependent Manner

**FIGURE 6:**
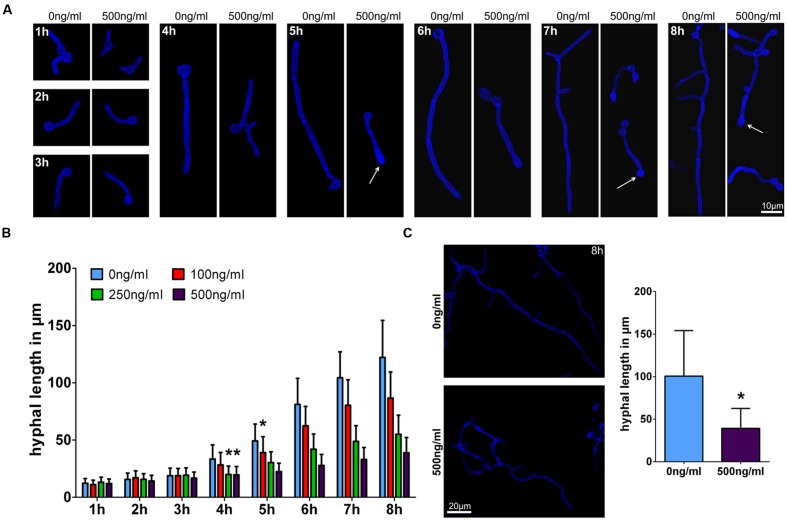
**Perforin affects hyphal elongation of *C. albicans*.** **(A)** *C. albicans* wild type cells were incubated in media (RPMI1640 + 5% heat-inactivated FBS) at 37°C without (control) or with 500 ng/ml perforin on a plastic surface for the indicated time points. After fixation and staining with calcofluor white, filaments were analyzed by microscopy. White arrows indicate swollen and yeast-like morphology aberrations. Data from one of at least three independent experiments with virtually identical results are shown. **(B)** Measurement of hyphal length over time. First incidence of hyphal elongation delay is marked with an asterisk. **(C)** After 8 h incubation of wild type *C. albicans* in SD + *N*-acetyl-D-glucosamine medium without (control) and with 500 ng/ml perforin, cells were subsequently fixed and stained with calcofluor white. Microscopic pictures and hyphal length measurements are shown. The bars show mean ± standard deviation of three independent experiments with *C. albicans* cells from different cultures. Estimation of *p*-values was performed with unpaired, two-sided Student’s *t*-test and asterisk indicates significance, ^∗^*p* ≤ 0.05.

Transcriptome data for NK cells confronted with *C. albicans* confirmed strong metabolic and functional activation of these immune cells. However, they did not reveal potential novel fungicidal effector mechanisms. Our previous studies have suggested that perforin is a major mediator of NK cell mediated anti-*C. albicans* activity (Voigt et al., 2014). To further analyze the effects of human perforin to hyphal growth, *C. albicans* yeast cells were incubated in the presence of different concentrations of human perforin. After 1–8 h fungal cells were fixed and morphology was analyzed microscopically. Although *C. albicans* yeast cells started to form germ tubes independent of perforin concentrations during the first hour of incubation, a significant reduction of hyphal length was observed after 4 h with perforin concentrations of 250 and 500 ng/ml compared to filaments grown without the substance (**Figures 6A,B**). For the lowest perforin concentration (100 ng/ml) tested this delay of hyphal elongation started after 5 h of treatment. Growth reduction increased with duration of hyphal induction. After 8 h filament lengths were reduced to 71% by 100 ng/ml, 45% by 250 ng/ml, and to 31% by 500 ng/ml perforin. Growth reduction correlated with morphology alterations showing swollen hyphal tips in the presence of perforin (**Figure 6A**, white arrows). To check if this observation was specific for serum-induced filamentous growth hyphal formation of *C. albicans* was additionally induced by *N*-acetylglucosamine containing media. A similar reduced elongation of *C. albicans* filaments as well as the swollen morphology by perforin could be observed under this condition (**Figure 6C**). Consequently, our results suggest that NK cell induced growth retardation of *C. albicans* was mainly mediated by perforin in a time- and concentration-dependent manner.

### The Transcriptional Response of *C. albicans* to Perforin

**FIGURE 7:**
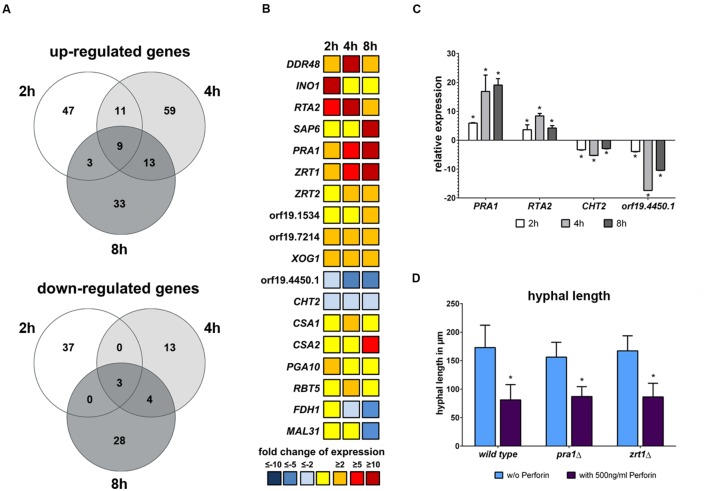
**Transcriptomic response of *C. albicans* to perforin shows an up-regulation of zinc homoeostasis-associated genes.** **(A)** The number of genes differentially expressed at least with a twofold change (*p* < 0.05) after treatment with perforin are shown in Venn diagrams for the three time points. **(B)** Dynamics of selected genes during the treatment with perforin. **(C)** qRT-PCR for four selected genes from B. Shown is the fold change of expression in media containing 500 ng/ml perforin compared to media alone. **(D)** *C. albicans* wild type and mutants lacking *PRA1* and *ZRT1* were grown at 37°C for 8 h without or with 500 ng/ml perforin. The hyphal length in all three strains is shown. The bars show mean ± standard deviation of three independent experiments with *C. albicans* cells from different cultures. Estimation of *p*-values was performed with unpaired, two-sided Student’s *t*-test and asterisks indicate significance, ^∗^*p* ≤ 0.05.

To identify the cause of morphological alteration and delay of hyphal elongation induced by perforin, the genome wide expression profile of *C. albicans* after exposure to this substance was analyzed by whole genome DNA microarrays. Fungal cells were incubated with or without 500 ng/ml perforin followed by RNA isolation at different time points. In total, only a limited set of genes showed differential expression in response to the addition of perforin: 110 genes after 2 h, 112 genes after 4 h and 93 genes after 8 h of perforin treatment (**Figure 7A**). Out of them, twelve genes were differentially expressed at all three time points, nine of those were up- and three were down-regulated. The response to perforin compromised the up-regulated genes *DDR48*, *RTA2*, *PRA1*, *ZRT1*, *XOG1*, orf19.6840, orf19.7214, orf19.4531, orf19.6601 and the down-regulated genes *CHT2*, orf19.2317, and orf19.4450.1 (**Figures 7A,B**; **Supplementary Table S3**). Expression patterns for four of these genes (*PRA1*, *RTA2*, *CHT2*, and orf19.4450.1) were verified by quantitative RT-PCR (**Figure 7C**).

#### Perforin Selectively Induces a Zinc Starvation Response in *C. albicans*

An interesting pattern was observed for genes involved in the maintenance of zinc homestasis in *C. albicans*. *PRA1* and *ZRT1* were found to be up-regulated during all time points with an increasing expression over time (**Figure 7B**; **Supplementary Table S3**). Both genes do not only share the same promoter, but were also shown to be important for sequestration of extracellular zinc ions (Citiulo et al., 2012). We have also observed an up-regulation of a second zinc transporter encoded by *ZRT2* at 4 h and 8 h time points (**Figure 7B**; **Supplementary Table S3**). At the 8 h time point, we noticed an additional up-regulation of the putative *C. albicans ZRT3* ortholog orf19.1534. The expression increase of these genes indicates that perforin might negatively influence zinc homeostasis in *C. albicans*. However, mutants lacking either *PRA1* or *ZRT1* showed the same hyphal elongation delay during perforin treatment as wild type filaments (**Figure 7D**). Due to the findings for zinc homeostasis, we looked for changes in transcriptomic responses involved in either calcium or iron homeostasis. Except for a slight up-regulation of the calcineurin-regulated transcription factor gene *CRZ1* at the 2 h time point (**Supplementary Table S3**) we did not observe clear changes for calcium maintenance. Regarding iron homeostasis, we noticed an up-regulation of the low iron-induced ferric reductase gene *FRE10* at 4 h, but genes which are normally increased under high iron conditions like orf19.2452 or *UCF1* were also up-regulated at the same time point (**Supplementary Table S3**). However, genes associated with iron utilization from heme and hemoglobin were partially up-regulated. This group included *CSA1*, *CSA2*, *PGA10*, and *RBT5* (**Figure 7B**), with *CSA2* being even strongly up-regulated at the 8 h time point with a more than eightfold increase.

#### NK Cells Do Not Suppress Expression of the Core Filamentation Response in *C. albicans*

Additionally, we noticed differential regulation of genes involved in cell wall remodeling. The exo-1,3-β-glucanase gene *XOG1* was up-regulated during all time points (**Figure 7B** **Supplementary Table S3**). The same pattern was observed for a yet uncharacterized gene, orf1.7214, which is putatively encoding a glucan-1,3-β-glucosidase (**Figure 7B**; **Supplementary Table S3**), indicating that perforin influences the assembly of 1,3-β-glucans within the fungal cell wall. On the other hand, the chitinase encoding gene *CHT2* was always down-regulated. The expression of the latter gene was confirmed by qRT PCR (**Figure 7C**).

At the 8 h time point we found a strong down regulation of *MAL31*, which is known to have an increased expression in HIV positive patients with oral candidosis (Zakikhany et al., 2007, **Figure 7B**; **Supplementary Table S3**). The most strongly down-regulated gene at this time point was *FDH1* (**Figure 7B**), encoding for a formate dehydrogenase. This gene was described to be repressed in yeast, but not hyphal cells (Doedt et al., 2004). Thus, its down-regulation might be a transcriptional correspondent to the perforin-induced lack of hyphal elongation. However, the majority of the previously defined core filamentation response genes (Martin et al., 2013) showed no changes compared to the control medium. The only exception was orf19.2457, with a slight down-regulation at the 4 h time point (**Supplementary Table S3**). Nonetheless, we did observe an up-regulation of hyphal growth repressor gene *NRG1* at 4 h, but not for the earlier and later time points (**Supplementary Table S3**). The up-regulation of the yeast cell-associated *SOD4* gene as well as the down-regulation of the aforementioned *FDH1* might indicate that a transcriptional transition from hyphal to yeast cell programs starting after 8 h.

## Discussion

Accumulating evidence suggests that NK cells are directly involved in antifungal immune responses. In animal models, depletion of NK cells resulted in enhanced fungal burden during *C. neoformans* infection, whereas adoptive transfer of NK cells mediated clearance of this fungal pathogen (Hidore and Murphy, 1986a,b; Schmidt et al., 2013). Reduced recruitment of NK cells to the infected lung in invasive aspergillosis due to depletion of MCP-1 (CCL2) resulted in a twofold greater mortality and increased fungal burden. Similarly, direct depletion of NK cells resulted in impaired protection and higher mortality (Morrison et al., 2003). For *C. albicans* systemic infection, depletion of NK cells has been shown to exert different effects depending on the host immune status. Depletion of NK cells in immunocompetent mice did not increase susceptibility to systemic candidiasis. On the contrary, NK cell-depleted mice were even found to be protected as a consequence of attenuated inflammation. However, depletion of NK cells in T/B/NK cell-deficient mice resulted in increased susceptibility to systemic *C. albicans* infection, which may point to an essential contribution of NK cells in immunocompromised hosts (Quintin et al., 2014). With NKp30 a unique NK cell receptor recognizing fungal pathogens has been identified (Li et al., 2013) and for most fungal pathogens analyzed so far, perforin seems to be the major mediator of NK cell mediated antifungal activity. The latter observation is amended by our analysis showing that perforin inhibits filamentation of *C. albicans* in a time and dose dependent manner and induces specific transcriptome adaptation in *C. albicans*. Currently it remains unclear to which extent direct antifungal activity of NK cells contributes to protective antifungal immunity, as NK cells are also an important source of both regulatory and pro-inflammatory mediators modulating other immune cells. Thus indirect effects of NK cells, e.g., on neutrophils or dendritic cells are likely an essential part of their role in the antifungal response network (Voigt et al., 2014). In the present study we have analyzed transcriptomic changes in NK cells after contact with *C. albicans*. Primary NK cells were analyzed in comparison to cytokine activated NK cells to reveal the full pattern of potential NK cell responses. These analyses revealed no qualitative differences between both cell types but suggested a more pronounced response of pre-activated cells to the fungal pathogen. KEGG analysis of differentially regulated genes highlighted the role of cytokine–cytokine receptor interactions in *C. albicans* activated NK cells. Among the genes found to be up-regulated were IFN-γ, GM-CSF, TNF-α, and MIP-1β (CCL4). Many studies in the past have further shown that NK specific cytokines such as IFN-γ and TNF-α can play an important role during *Candida* infections (Djeu et al., 1986, 1988; Ferrante, 1989; Maródi et al., 1993). Our findings further corroborate the immunomodulatory potential of NK cells via secretion of cytokines (Ferrante, 1989; Costantini et al., 2010). Up-regulation of components of the MAPK signaling pathway indicated an activation of the immune cells, as this signaling pathway is known to contribute to NK cell cytotoxicity (MacFarlane and Campbell, 2006). In response to the confrontation with *C. albicans*, primary and cytokine activated NK cells up-regulated several genes encoding key enzymes of the glycolysis. It has been shown that glycolytic metabolism is crucial for development and function of NK cells (Marcais et al., 2014). Indeed, glycolysis is important for several lymphocyte populations and is integrally linked to their differentiation and function (Donnelly and Finlay, 2015). Cytokine dependent activation of NK cells correlated with a glycolytic reprogramming, which triggered the up-regulation of glycolytic enzymes and glucose transporters (Donnelly et al., 2014). The major effector molecules of NK cells, perforin and granzyme B can also be regulated by glycolytic reprogramming (Finlay et al., 2012; Donnelly et al., 2014; Marcais et al., 2014). Indeed, experiments using the glycolysis inhibitor 2-DG in the confrontation of NK cells with *C. albicans* clearly showed that inhibition of glycolysis reduces down-regulation of CD16 and release of perforin and GM-CSF. In contrast, no effects of 2-DG could be detected for up-regulation of CD107a and release of IFN-γ, indicating specific contributions of glycolysis to NK cell activation. This is in line with data showing that glycolysis dependent NK cell activation is dependent on the mTORC1 signaling pathway (Donnelly et al., 2014; Marcais et al., 2014). Within our transcriptome data we observed an up-regulation of genes encoding for c-Myc and hypoxia-inducible factor 1α (HIF1α) in *C. albicans* infected cytokine activated NK cells, both transcription factors controlled by mTORC1 that regulate the expression of multiple glycolytic enzymes (Finlay, 2015). Together with the c-Myc transcription factor, mTORC1 is also involved in glycolytic activation in T cells and B lymphocytes (Wang et al., 2011; Powell et al., 2012; Sinclair et al., 2013; Caro-Maldonado et al., 2014; Oestreich et al., 2014; Donnelly and Finlay, 2015). We have not yet addressed the NK cell receptor(s) that are responsible for mediating *C. albicans* induced activation. However, a recent study identified NK cell receptor NKp30 to be responsible for fungal recognition and killing (Li et al., 2013). As activation of NKp30 is required for perforin release in response to fungal pathogens and blocking of glycolysis also resulted in diminished secretion of perforin in our experiments (see **Figure 5**), NKp30 signaling might be involved in regulation of glycolysis during *Candida* infection.

As perforin is the major secreted NK cell protein (Voigt et al., 2014), it was subject of a more detailed analysis focusing on its consequences for the fungal pathogen. Exposure to perforin led to a concentration dependent growth arrest during hyphal elongation. The fungal transcriptomic response was characterized by up-regulation of genes involved in zinc homeostasis (*PRA1*, *ZRT1*, *ZRT2*, *ZRT3*). So far, two of these genes were shown to be crucial to sequester environmental zinc (Citiulo et al., 2012). However, neither a *pra1*Δ nor a *zrt1*Δ mutant responded different from wild type when confronted with perforin. On the other hand, several works from the past indicate that zinc could indeed be helpful for the fungus to encounter the effects of perforin. Decades ago it was shown that zinc can inhibit the functions of streptolysins (Avigad and Bernheimer, 1978). As streptolysins and perforin share structural and functional similarities (Gilbert, 2010), it is possible that zinc can also prevent the negative effects of perforin treatment. Additionally, zinc ions participate in the closure of cytolysin-caused channels in Lettre cells (Bashford et al., 1988) or pores formed from either alpha-hemolysin (Walker et al., 1995) or pneumolysin (Franco-Vidal et al., 2008). Based on these previous results, we suggest that zinc homeostasis might be involved in the response of *C. albicans* to perforin treatment, although the details remain to be elucidated. In addition to zinc homeostatis, genes involved in the heme/hemoglobin iron utilization were found to be up-regulated although to a lesser extent than the zinc-associated genes. Transcript levels of *CSA2* were highly increased at the 8 h time point. The strong induction of the gene might be linked to perforin-induced reduction of iron availability, especially as Csa2 is speculated to act as a kind of “hemophore” (Crawford and Wilson, 2015). However, *CSA2* was also shown to be up-regulated in response to other stressful environments, for example the presence of nitric oxide (Hromatka et al., 2005). The most down-regulated gene during co-incubation with perforin at all three time points was orf19.4450.1. The gene encodes a small protein of 68 amino acids without known function. However, previous transcriptome studies showed an up-regulation of the gene transcript during fungal growth in the intestinal tract and biofilm formation (Rosenbach et al., 2010; Bonhomme et al., 2011), which is in line with the reduced gene expression of orf19.4450.1 during perforin-induced inhibition of *C. albicans* filamentation.

Taken together, our data confirm activation of NK cells by *C. albicans* and show that beside functional activation metabolic adaptation takes place by increased glycolysis. Furthermore we describe the induction of a *bona fide* zinc depletion response in a fungal pathogen after exposure to perforin, suggesting a potential link of perforin action to this essential micronutrient.

## Author Contributions

DH, JV, KH, MB, RM performed experiments; SB contributed reagents and mutants; DA-E, MW analyzed data; KH, JL, RM, OK designed experiments and planned study; DH, JV, KH, RM, OK wrote the manuscript; MB, JL, DA-E, MW, SB critically revised the manuscript; DH and JV contributed equally to this manuscript.

## Conflict of Interest Statement

The authors declare that the research was conducted in the absence of any commercial or financial relationships that could be construed as a potential conflict of interest.

## Footnotes

1 http://www.r-project.org

2 http://david.abcc.ncifcrf.gov/

## Supplementary Material

**TABLE S2 | Differentially expressed genes of primary and cytokine activated NK cells confronted with *C. albicans*.**

**TABLE S3 | Differentially expressed genes of perforin-treated *C. albicans*.**
