# Supplementary material for: Candida albicans Induces Metabolic Reprogramming in Human NK Cells and Responds to Perforin with a Zinc Depletion Response
Source: Front Microbiol. 2016 May 19;7:750. doi: 10.3389/fmicb.2016.00750 (PMC4872603; doi:10.3389/fmicb.2016.00750)
Supplement: Supplementary file 3 [file Table_1.DOCX]

**Table S1. Oligonucleotide primers used in this study.**

| **oligonucleotide** | **sequence in 5'-3' direction** |
| --- | --- |
| R1-CaRTA2 | CTATAGAGTGGCAGAATTGGCTG |
| R2-CaRTA2 | GTATGTCATTTGCAACCACGCTAGA |
| R1-CaCHT2 | GTATGCACCGAAGAAACATGTGTTC |
| R2-CaCHT2 | GTAACTGGGGTAATAGTAGAAGTGAG |
| R1-19.4450.1 | TACTTACTCCTGGTACTGCTATATTAATTG |
| R2-19.4450.1 | CATTGAGATTTGTAGTTACCTTCATACTG |
| R1-CaPRA1 | ATGGAGATGGTACTCTGTACAAG |
| R2-CaPRA1 | CAGTGGACTTCACCATCTGC |
| R1-CaACT1 | TCAGACCAGCTGATTTAGGTTTG |
| R2-CaACT1 | GTGAACAATGGATGGACCAG |
| R1-ECE1 | ATCGAAAATGCCAAGAGAG |
| R2-ECE1 | AGCATTTTCAATACCGACAG |
| R1-UME6 | TCTACTTCTAATCCAATGGTG |
| R2-UME6 | TATCATTACTTGATTTTTTCCGAG |
